# Large-scale plasmidome and carbapenem mobilome analysis reveals a mechanistic duality: high-power specialists and structural generalists mobile genetic elements

**DOI:** 10.3389/fmicb.2026.1755819

**Published:** 2026-02-20

**Authors:** Hannay Crystynah Almeida de Souza, Anamaria M. P. dos Santos, Arlen Carvalho de Oliveira Almeida, Ana Beatriz Portes, Carlos Adam Conte-Junior, Pedro Panzenhagen

**Affiliations:** 1 Center for Food Analysis (NAL), Technological Development Support Laboratory (LADETEC), Federal University of Rio de Janeiro (UFRJ), Cidade Universitária, Rio de Janeiro, Brazil; 2 Laboratory of Advanced Analysis in Biochemistry and Molecular Biology (LAABBM), Department of Biochemistry, Federal University of Rio de Janeiro (UFRJ), Cidade Universitária, Rio de Janeiro, Brazil; 3 Graduate Program in Biochemistry (PPGBq), Institute of Chemistry (IQ), Federal University of Rio de Janeiro (UFRJ), Cidade Universitária, Rio de Janeiro, RJ, Brazil; 4 Graduate Program in Veterinary Hygiene (PGHIGVET), Faculty of Veterinary Medicine, Fluminense Federal University (UFF), Niterói, RJ, Brazil; 5 Graduate Program in Food Science, Institute of Chemistry, Federal University of Rio de Janeiro, Rio de Janeiro, RJ, Brazil; 6 Laboratory of Microorganism Structure, Department of General Microbiology, Institute of Microbiology Paulo de Góes (IMPG), Federal University of Rio de Janeiro, Rio de Janeiro, Brazil; 7 Analytical and Molecular Laboratory Center (CLAn), Institute of Chemistry (IQ), Federal University of Rio de Janeiro (UFRJ), Cidade Universitária, Rio de Janeiro, Brazil

**Keywords:** antimicrobial resistance, carbapenemase dissemination, MGEs, multidrug-resistance plasmids, transposable elements

## Abstract

**Introduction:**

Carbapenem resistance represents a critical global public health threat, with the rapid dissemination of carbapenemase genes largely mediated by plasmids. Although mobile genetic elements (MGEs), including transposons (Tn), insertion sequences (IS), and integrons (In), are known to drive this process, the mechanistic interplay between different MGE classes and the carbapenem resistome remains insufficiently characterized at a global scale.

**Methods:**

We performed a large-scale, systematic analysis of 6,017 plasmids carrying carbapenemase genes extracted from 72,556 complete plasmid sequences. Antimicrobial resistance genes were identified using AMRFinderPlus, and MGEs were detected through an integrative workflow combining TnCentral, INTEGRALL, ISFinder, ABRicate, and ISEScan. Functional associations between carbapenemase genes and MGEs were defined using a conservative genomic proximity threshold (≤5 kb). Statistical association analyses, network topology metrics, and a Mobilization–Resistance Score were applied to characterize mobilization strength and replicon-level dissemination potential.

**Results:**

Our analysis revealed a dual mechanistic architecture governing carbapenem resistance dissemination. Integrons and transposons acted as high-fidelity specialists, showing the strongest associations with carbapenemase genes (median ORs: 107.2 and 92.7, respectively). In contrast, insertion sequences exhibited lower individual association strength (median OR: 17.3) but structured the global mobilome network. IS6_292 emerged as the apex generalist, displaying the highest betweenness centrality (3,815.87) and forming significant associations with 13 distinct carbapenemase genes. At the replicon level, dissemination potential quantified by the Mobilization–Resistance Score identified IncFIB(pQil) and IncR as the most potent supervector platforms, despite the higher frequency of IncX3 and IncL plasmids. These replicons combine high MGE diversity with multiple carbapenemase genes, forming high-risk vectors for resistance acquisition and spread.

**Discussion:**

These findings demonstrate that carbapenemase dissemination is driven by complementary mobilization strategies: structurally specialized, high-strength transmission mediated by integrons and transposons, and structurally central, generalist dissemination mediated by insertion sequences. This duality reshapes the understanding of carbapenem resistome evolution and highlights structurally critical MGEs and high-scoring plasmid replicons as strategic surveillance targets. Focusing genomic monitoring on these elements may support the development of precision-based containment strategies to mitigate the global spread of carbapenem resistance.

## 1 Introduction

Antimicrobial resistance (AMR) is one of the most urgent challenges to global public health in the 21st century, progressively compromising the therapeutic efficacy of last-resort antimicrobials (World Health Organization, 2025). Carbapenem-resistant bacteria, particularly *Enterobacterales* and *Acinetobacter baumannii*, are classified by the World Health Organization (WHO) as critical-priority pathogens in the WHO bacterial priority pathogens list. This classification enhances the urgent need for the development of new antibiotics in response to the increasing spread of these strains, which limits the available therapeutic options (World Health Organization, 2024).

Plasmids are central vectors in the dissemination of carbapenem resistance and the spread of multidrug-resistant pathogens (de Souza et al., 2025a). These extrachromosomal elements are classified into incompatibility (Inc) families, defined by the similarity of their replication and partitioning regions, which determine their replicative identity and ability to coexist within the same host cell (Novick, 1987; Watts et al., 2022). Plasmids from the same family do not remain stable within the same bacterium due to competition for replication systems, which explains the replicative exclusivity between plasmid families and shapes the distribution and persistence of resistance genes (Darphorn et al., 2021). Previously, significant associations between specific Inc groups and carbapenemase genes were detected, including IncL, ColKP3, IncM2, IncC, IncFII(pHN7A8), IncR, and IncFII with *bla*_OXA-48_, *bla*_OXA-181_, *bla*_IMP-1_, *bla*_NDM-4_, *bla*_KPC-2_, *bla*_KPC-2_, and *bla*_NDM-5_, respectively, emphasizing the role of these plasmids as critical epidemiological vectors in the dissemination of carbapenem resistance (de Souza et al., 2025a).

Other mobile genetic elements (MGEs), including transposons (Tn), insertion sequences (IS), and integrons (In), are frequently found in plasmids and act as amplifiers of AMR by promoting recombinations and rearrangements that mobilize resistance genes across phylogenetically diverse bacterial species (Partridge et al., 2018). These mechanisms accelerate the spread of carbapenemases and facilitate the emergence of highly adaptive multidrug-resistant strains, enabling the persistence in diverse clinical and ecological environments (Shropshire et al., 2022; Fang et al., 2024; Han et al., 2025).

Despite the recognized role of MGEs in the mobility of the carbapenem resistome, their interdependence and functional interactions within plasmid backbones remain poorly explored. Understanding how plasmids, transposons, insertion sequences, and integrons cooperate to mobilize, amplify, and stabilize carbapenemase genes is important for elucidating the mechanisms that enable the rapid emergence and global dissemination of multidrug-resistant lineages. This knowledge gap camouflages the ability to predict the evolutionary trajectories of high-risk clones and limits the development of surveillance markers that can anticipate the spread of clinically relevant resistance determinants.

Systematically characterizing the plasmid mobilome associated with carbapenemases and unveiling the network of MGEs that structure these resistance loci provides a comprehensive acknowledgement to identify the relevant genetic vectors that sustain the global expansion of carbapenem resistance. This study’s findings provide unprecedented molecular insights that can support enhanced genomic surveillance programs, inform the design of targeted containment strategies, and guide the development of next-generation antimicrobial and anti-plasmid therapeutics.

## 2 Materials and methods

### 2.1 Genomic data collection and identification of antimicrobial resistance genes

On June 25, 2025, a total of 72,556 complete plasmid sequences were extracted from the Plasmid database (PLSDB) (version 2024_05_31_v2), a comprehensive plasmid repository of the National Center for Biotechnology Information (NCBI). PLSDB applies systematic quality control procedures, including sequence filtering and deduplication, ensuring that each record represents a unique complete plasmid sequence.

AMR genes were detected in the 72,556 plasmid sequences using the AMRFinderPlus tool (version 3.12.8), based on standard parameters of at least 90% identity and 60% coverage. Among these, 28,836 sequences presented at least one AMR gene. The resulting data were subsequently integrated with data from mobile genetic elements, allowing analysis of the association between AMR genes and the mobilome of plasmid sequences.

### 2.2 Detection of mobile genetic elements

The identification of MGEs was performed using an integrative approach that combined two complementary tools. Initially, we performed the search using the TnCentral BLAST library, integrating TnCentral, INTEGRALL, and ISFinder^1^ with ABRicate^2^ (version 1.0.1) to apply these tools to our sequences. To increase detection sensitivity and identify elements not recognized by the TnCentral database (Ross et al., 2021), we additionally used the ISEScan (version 1.7.2.3) (Xie and Tang, 2017). Subsequently, all results were compiled into a single standardized annotation table using an R-based integration workflow. The complete integrated dataset is provided as Supplementary Table S7 (Merged_MGE_Annotations), and the R script used for data integration is available as Supplementary File S1.

### 2.3 Detection of mobile genetic elements associated with antimicrobial resistance genes

The association between AMR genes and MGEs was performed with a maximum proximity of 5 kilobases (kb), as defined by the VR Profile2 tool and described by Shang et al. (2025). Furthermore, to identify potential compound transposition events, we considered carbapenemase genes flanked by identical insertion sequences at a maximum distance of 5 kb from each gene boundary. This criterion was applied to infer genomic contexts compatible with classical compound transposon structures. This approach allows the detection of possible genomic arrangements consistent with classical compound transposon structures, thus increasing the accuracy of the inferred mobilization mechanisms (Wang et al., 2022; Shang et al., 2025).

### 2.4 Extraction of plasmid incompatibility groups

Plasmid Inc group data were obtained from the PLSDB, which provides curated plasmid sequence metadata, including replicon typing results from the PlasmidFinder database. These annotations were used to identify plasmid replicons across deposited sequences accurately.

### 2.5 Identification of plasmid supervectors

For each plasmid carrying at least one mobilization element associated with carbapenemase genes, we quantified (i) the number of distinct mobilization elements and (ii) the number of unique carbapenemase genes present. To explore which plasmids combined the greatest mobilization capacity with resistance diversity, we computed the simple product between these two variables:


$$\begin{array}{l} \text{Mobilization}-\text{resistance score}=(\begin{array}{l} \text{Number of unique}\;\\ \text{mobilization elements} \end{array})\times \\ (\begin{array}{l} \text{Number of unique}\;\\ \text{carbapenemase genes} \end{array}) \end{array}$$

This score was used exclusively as an operational criterion within this study to highlight plasmids that accumulated higher values, interpreted as having a greater potential to disseminate carbapenemase genes. Plasmids with the highest scores were therefore referred to as “supervectors,” reflecting their combined mobilization and resistance-loading characteristics. Finally, plasmids were grouped according to their incompatibility (Inc) families, allowing us to identify which plasmid groups contained the greatest concentration of high-scoring plasmids.

### 2.6 Statistical analysis

#### 2.6.1 Gene-carrying capacity and outlier treatment

The ability of IS, Tn, and In to carry carbapenem resistance genes was evaluated by quantifying, for each element, the number of distinct carbapenemase genes associated with it. Because the frequency of MGEs was significantly different, each MGE was analyzed individually, without normalization, allowing the results to accurately represent the true diversity of genes associated with each element, avoiding bias. To minimize distortion caused by exceptionally high or low values, statistical outliers were identified using the interquartile range (IQR) method. The IQR was defined as the range between the 25th percentile (Q1) and the 75th percentile (Q3). Values falling below Q1 − 1.5 × IQR or above Q3 + 1.5 × IQR were excluded from the analysis. Because the distribution of gene counts is typically skewed by a few highly mobilizing elements, the median was selected as the central measure, ensuring a robust representation of the typical gene-carrying profile for each MGE type.

#### 2.6.2 Association between carbapenem resistance genes and mobilization elements

Carbapenemase-MGE association data were obtained from three mobile genetic element categories: In, IS, and Tn For each AMR-MGE pair within each category, 2 × 2 contingency tables were constructed based on co-occurrence patterns across all sequences, where *a* = sequences containing both AMR and MGE, *b* = sequences with AMR but not MGE, *c* = sequences with MGE but not AMR, and *d* = sequences with neither element. Odds ratios (OR) were calculated as OR = (*a* × *d*)/(*b* × *c*), with Haldane’s correction applied when zeros occurred: OR = ((*a* + 0.5) × (*d* + 0.5))/((*b* + 0.5) × (*c* + 0.5)). Statistical significance was assessed using Fisher’s exact test, with *p*-values adjusted for multiple comparisons using the Benjamini–Hochberg false discovery rate (FDR) method. Significant associations were defined as FDR <0.05 (or FDR <0.01 for the top 30 ranking), OR >1 (or OR ≥3 for stringent filtering), and a minimum of 3–5 co-occurrences. Jaccard similarity index (*J* = *a*/(*a* + *b* + *c*)) was calculated to measure the overlap between AMR and MGE occurrence patterns.

For the purposes of statistical association analysis, an AMR-MGE pair was considered positively associated only when both elements occurred within a maximum genomic distance of 5 kb, consistent with criteria used to infer functional mobilization events. Plasmid sequences containing both an AMR gene and an MGE separated by distances greater than 5 kb were not considered functionally associated and were treated as non-associated in the construction of contingency tables. This conservative definition was adopted to minimize associations driven by simple co-residence on the same plasmid backbone and to ensure that statistical inferences reflect biologically meaningful mobilization contexts.

Mobile genetic elements were classified by specificity based on the number of distinct carbapenemases with which they formed significant associations: specialists (1 carbapenemase), intermediate (2–3 carbapenemases), and generalists (≥8 carbapenemases). For network analysis, a bipartite graph was constructed with carbapenemases and MGEs as nodes and significant associations (FDR <0.05) as edges weighted by OR values. Network centrality metrics were calculated using the *igraph* package, including degree centrality (the number of direct connections), betweenness centrality (the frequency at which a node appears on the shortest paths between other nodes), and PageRank (an importance score based on the network structure with a damping factor of 0.85). Pairwise comparisons of OR distributions between MGE categories were performed using Wilcoxon rank-sum tests on log₁₀-transformed values. All statistical analyses were performed in R (version 4.4.3).

## 3 Results

### 3.1 Resistome profiling of carbapenem-resistant sequences

Among the 28,836 resistant plasmid sequences, 6,017 (20.9%) contained at least one gene encoding carbapenem resistance. The most frequently resistance genetic variants were *bla*_KPC-2_ (*n* = 1,513, 25.1%), *bla*_NDM-1_ (*n* = 1,006, 16.7%), *bla*_NDM-5_ (*n* = 770, 12.8%), *bla*_OXA-48_ (*n* = 672, 11.2%), *bla*_KPC-3_ (*n* = 454, 7.5%), *bla*_VIM-1_ (*n* = 187, 3.1%), *bla*_OXA-181_ (*n* = 169, 2.8%), *bla*_IMP-4_ (*n* = 151, 2.5%) and *bla*_IMP-1_ (*n* = 143, 2.4%).

In the analysis of carbapenemase genes, which encode carbapenemase enzymes of different classes, class B enzymes were the most frequent, present in (*n* = 2,677, 44.5%) of the sequences, followed by class A (*n* = 2,039, 33.9%) and class D (*n* = 1,305, 21.7%) enzymes. Individual plasmid resistance load varied from one to two distinct carbapenemase genes, with the median showing a predominant carriage of only one distinct gene per sequence. While a small fraction of plasmids carried two distinct genes, the co-occurrence of multiple carbapenemase genes on the same plasmid was uncommon (*n* = 136, 2.2%).

### 3.2 Characterization of the mobilome in plasmids carrying carbapenem resistance genes

**Figure 1:**
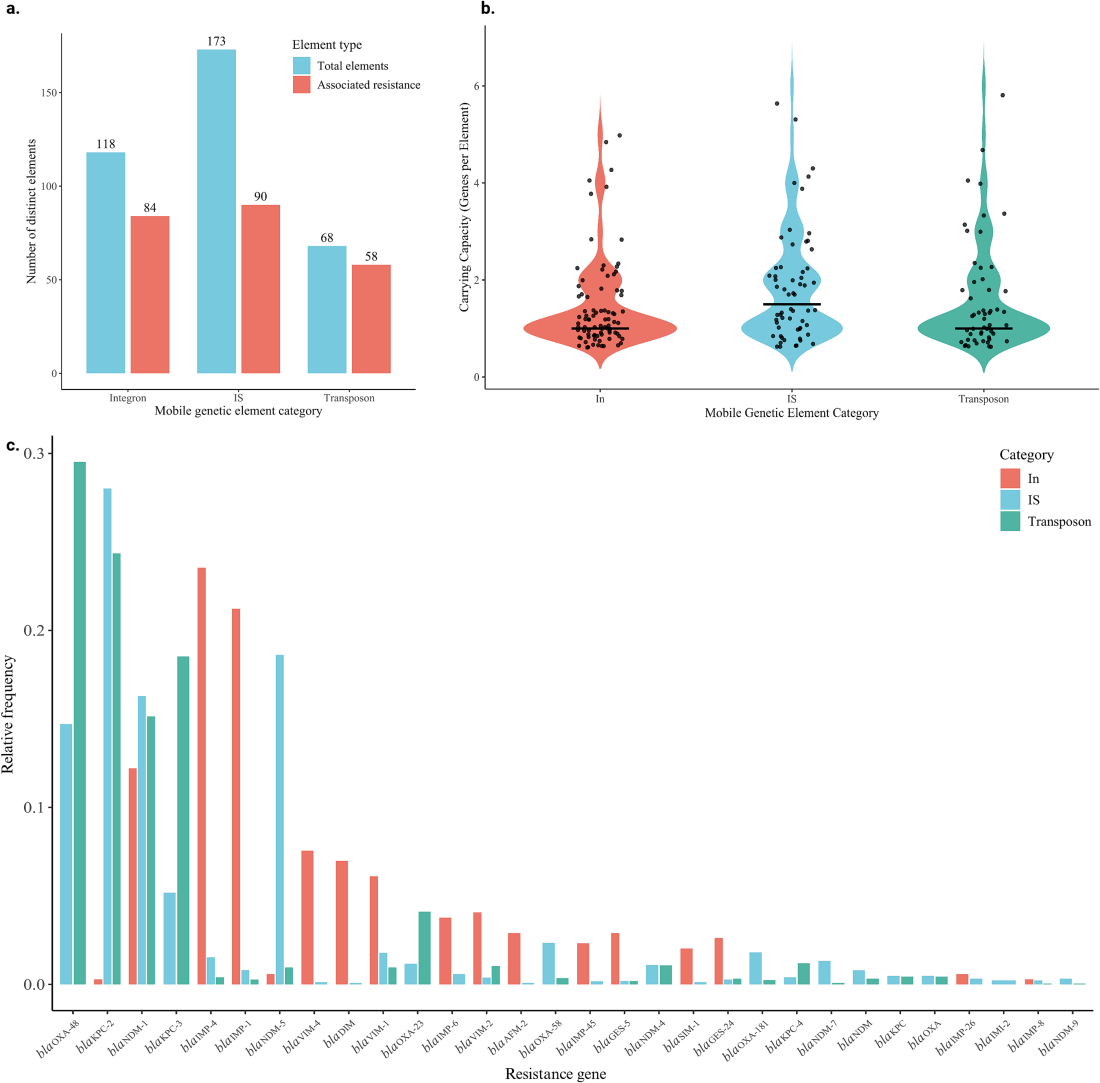
Characterization of mobile genetic elements (MGEs) and their association with carbapenem resistance genes. **(a)** Number of distinct MGEs identified in plasmid sequences and their association with carbapenem resistance genes. The blue bars represent the total number of elements in each category, and red bars indicate the subset of elements found in proximity to carbapenem resistance determinants. **(b)** Distribution of resistance gene carrying capacity by mobilization elements. Each point represents an individual element, while the black line indicates the median. **(c)** Distribution of the 30 most frequent variants of carbapenem resistance genes among different categories of mobilization elements. Bars represent the relative frequency of AMR-MGE association events within each mobilization category, calculated independently for transposons (Tn), integrons (In), and insertion sequences (IS). For each category, relative frequencies were normalized by the total number of association events observed within that category, considering only the 30 most abundant carbapenemase genes in the entire dataset. Multiple associations involving the same resistance gene were counted as independent events.

Analysis of plasmid sequences identified 173 IS, 68 Tn, and 118 In elements. Of these, 90/173 (73.4%) IS, 58/68 (85.3%) Tn, and 84/118 (71.2%) In elements were found in proximity to carbapenem resistance determinants, based on predefined association criteria (Figure 1a). The high frequency of association of carbapenemase genes with these mobile genetic elements demonstrates how these modules contribute to maintaining carbapenem resistance in plasmids.

The transport capacity of different carbapenem resistance variants was evaluated among the mobile genetic elements present in the plasmid sequences (Figure 1b), showing that IS carries a greater diversity of genes per element compared to Tn and In (Figure 1b). The medians confirmed that IS had the highest transport capacity, although the differences between categories were relatively small.

The distribution of mobilization categories was observed among the 30 most frequent carbapenemase gene variants (Figure 1c). Among the most prevalent genes, *bla*_OXA-48_ stood out due to its high frequency of association with Tn, followed by *bla*_KPC-3_. In contrast, different patterns were observed for *bla*_KPC-2_, *bla*_NDM-1_, and *bla*_NDM-5_, which were preferentially associated with IS. Conversely, *bla*_IMP-4_ and *bla*_IMP-1_ showed a higher frequency of association with In. It can be observed that some genes, even belonging to the same subclass, can opt for different mobilization mechanisms and, consequently, different dissemination pathways.

#### 3.2.1 Quantification of key MGEs in carbapenem resistance dissemination

To identify the MGEs most relevant for the dissemination of carbapenemase genes, the number of mobile elements associated with carbapenemase genes was quantified. Among the 25 MGEs most frequently associated with carbapenem resistance, IS were the most frequent (18/25, 72.0%), followed by Tn (6/25, 24.0%) and a single In (1/25, 4.0%).

**Figure 2:**
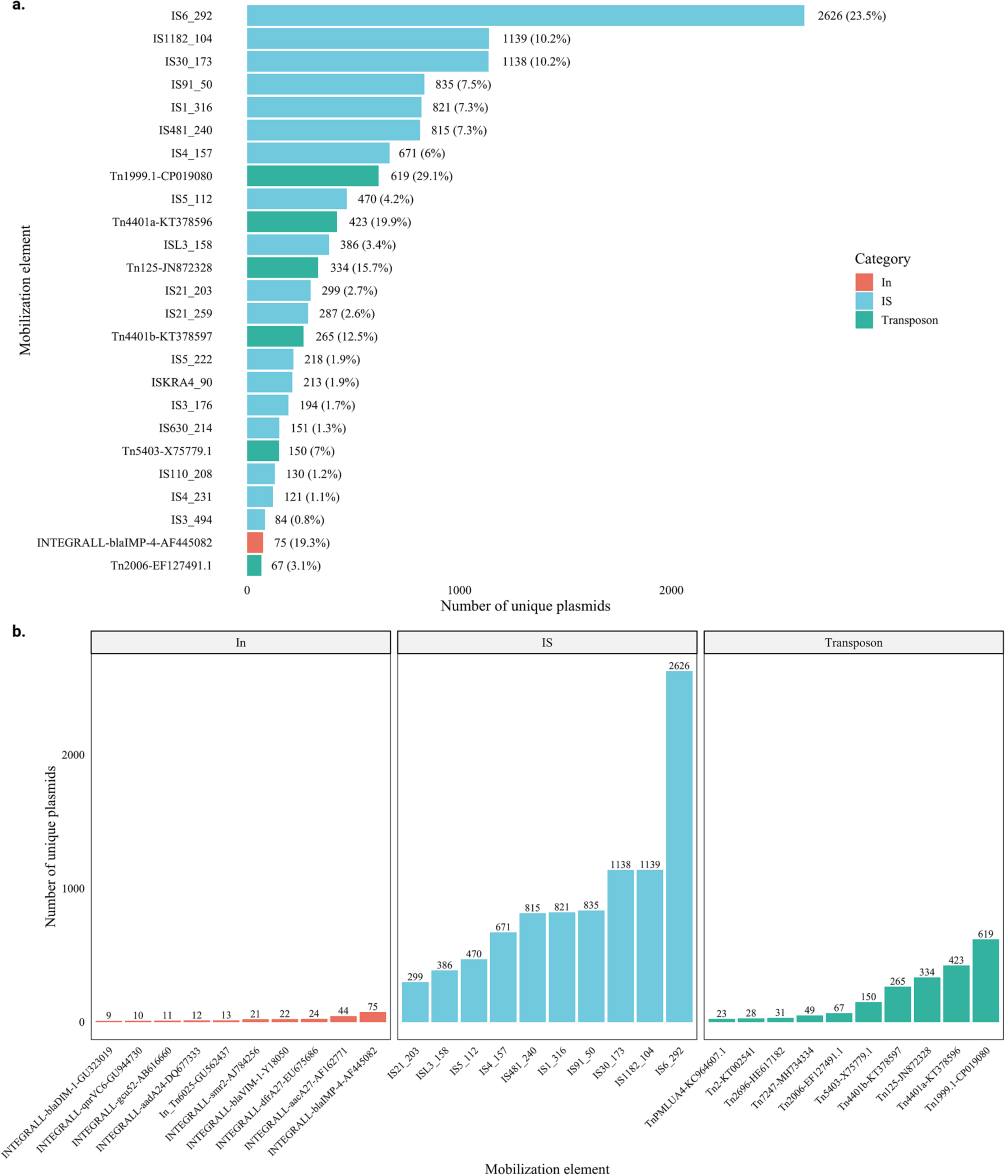
Distribution of mobile genetic elements (MGEs) associated with carbapenemase genes. **(a)** Proportion of the 25 most frequent MGEs linked to carbapenem resistance. **(b)** Top 10 most frequently associated MGEs within each mobilization category (IS, transposons, integrons).

Among the 10 most frequently associated with carbapenemases, IS6 (2,626, 23.5%) was the most prominent, followed by IS1182 (1,139, 10.2%) and IS30 (1,138, 10.2%). In the case of Tn, the most common were Tn1999.1 (619, 29.1%), followed by Tn4401a (423, 19.9%), and Tn125 (334, 15.7%) (Figure 2a). The INTEGRALL-*bla*_IMP-4_-AF445082 was the most frequent (75, 19.3%) and, along with the other integrons, accounted for only a small fraction among all the MGEs. The distribution of the 10 most frequent mobilization elements within each category is shown in Figure 2b.

#### 3.2.2 High-frequency composite transposons harboring carbapenemase genes

**Figure 3:**
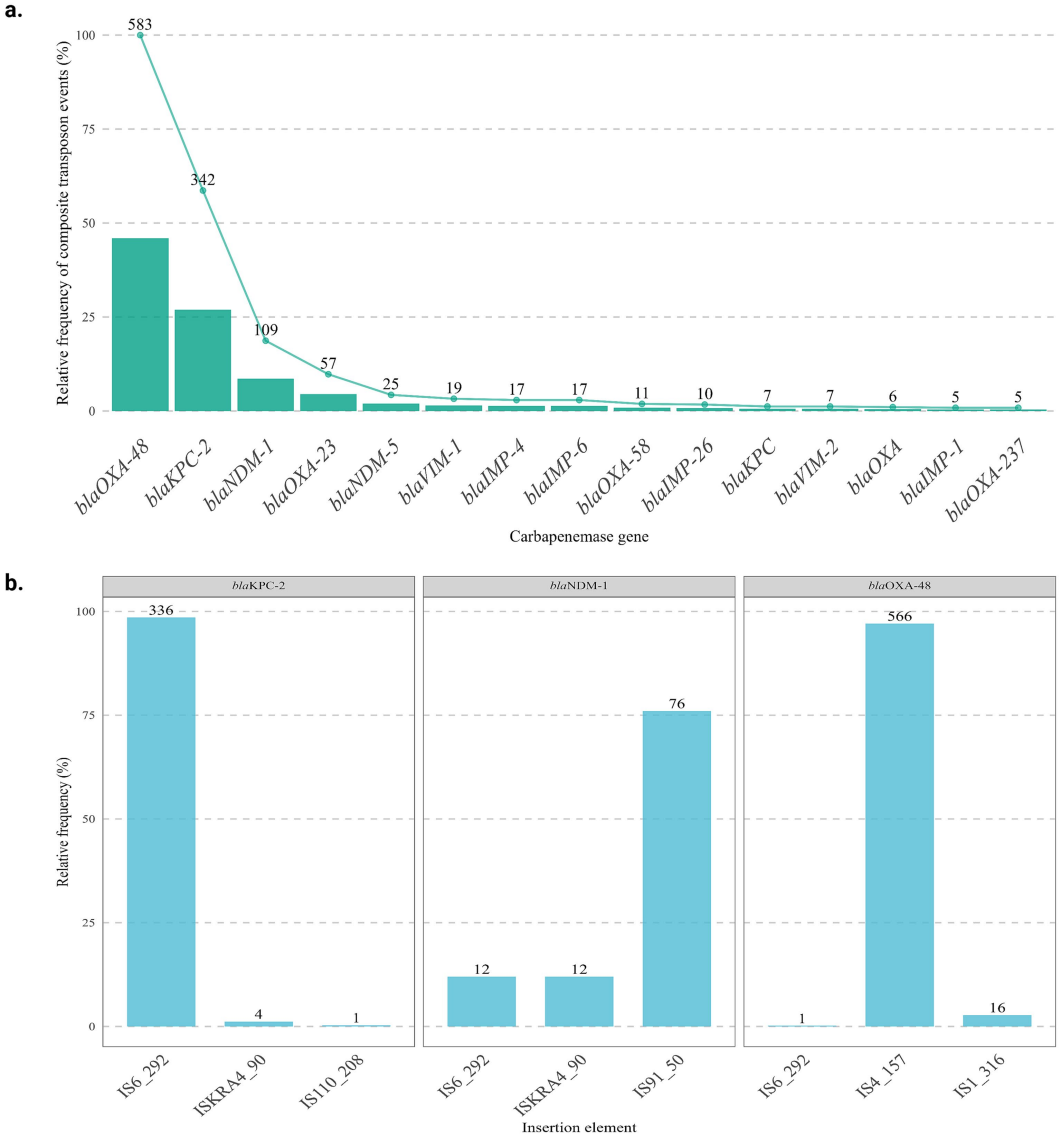
Distribution of composite transposon events among carbapenemase genes. **(a)** Relative frequency and absolute number of composite transposon events across the 15 most prevalent carbapenemase genes. **(b)** Distribution of the major insertion sequence (IS) elements associated with composite transposons carrying the three carbapenemase genes with the highest mobilization frequency.

A total of 1,268 compound transposition events involving different carbapenemase gene variants were identified, corresponding to 22.8% of the total occurrences observed between IS and carbapenemase genes (*n* = 1,268/5,322). The relative distribution of these events was highest for the *bla*_OXA-48_ variant, followed by *bla*_KPC-2_ and *bla*_NDM-1_. Together, these three variants accounted for more than 80% of all detected events (Figure 3a). Next, the main IS responsible for mobilization of these genes was analyzed, and the most frequent vectors associated with composite Tn identified in each gene were IS4_157 for *bla*_OXA-48_, IS6_292 for *bla*_KPC-2_, and IS91_50 for *bla*_NDM-1_ (Figure 3b).

### 3.3 Co-occurrence of mobilization elements among carbapenemase genes

**Figure 4:**
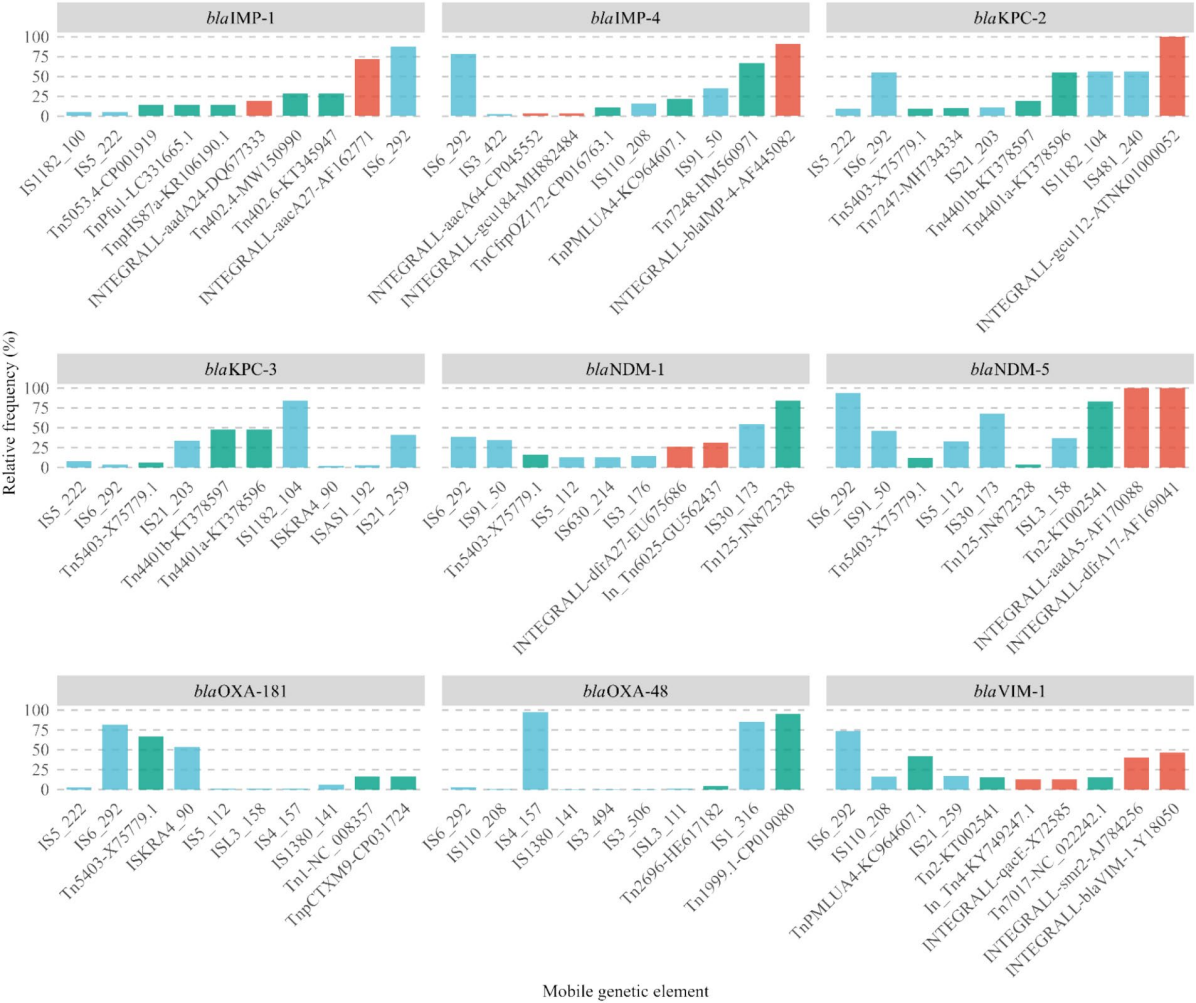
Ten most frequent mobilization elements in the main carbapenemase genes. Colors represent the element category: blue = insertion sequences, green = transposons, red = integrons. The relative frequency of mobilization elements was calculated within each category.

The distribution of the top 10 MGEs was analyzed among the most prevalent carbapenemase gene variants, including *bla*_KPC-2_, *bla*_NDM-1_, *bla*_NDM-5_, *bla*_OXA-48_, *bla*_KPC-3_, *bla*_VIM-1_, *bla*_OXA-181_, *bla*_IMP-4_, and *bla*_IMP-1_, which together represent approximately 80% of all identified variants (see Figure 4).

### 3.4 Association between carbapenem resistance genes and mobilization elements

To comprehensively map the mobilome landscape in carbapenemase dissemination, we performed a systematic paired-association analysis on 18,878 plasmid sequences, that carried at least one antimicrobial resistance (AMR) gene located within a genomic distance of ≤5 kb from a mobile genetic element, irrespective of resistance class. This dataset comprised 845 carbapenemase genes and 209 mobile genetic elements (MGEs) distributed among integrons (In), insertion sequences (IS), and transposons (Tn). This large-scale analysis identified 1,323 significant associations (FDR <0.05), revealing remarkable hierarchies in both association strength and mechanistic specificity. The strongest associations between carbapenemases and MGEs exhibited extraordinary mobilization potency, with odds ratios reaching very high magnitudes.

**Figure 5:**
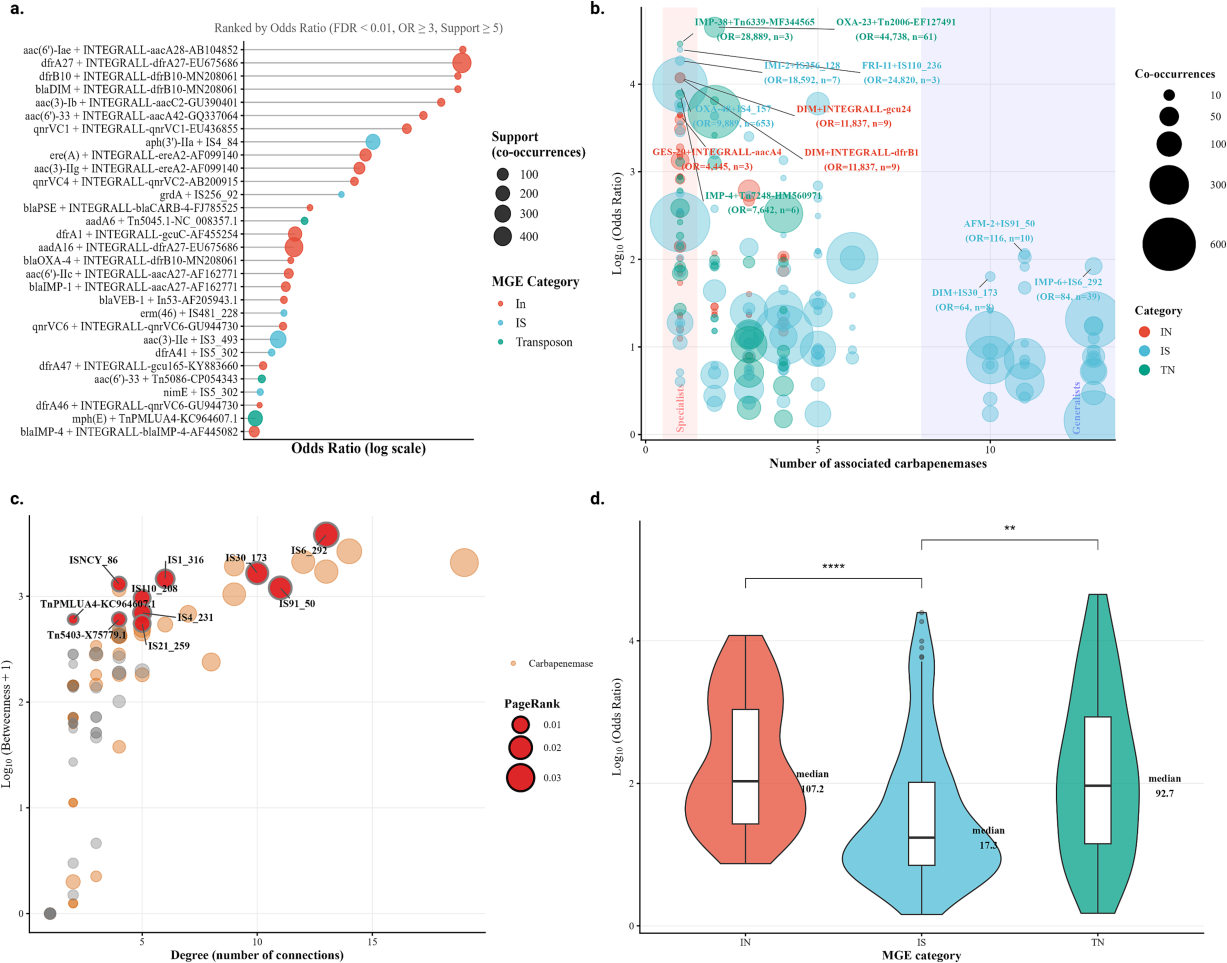
Association between carbapenem resistance genes and mobilization elements. **(a)** Top 30 significant associations between carbapenemase genes and mobile genetic elements (MGEs), ranked by odds ratio (FDR <0.01, OR ≥3, support ≥5). Node size indicates the number of co-occurrences (support), and colors represent MGE categories: integrons (In, red), insertion sequences (IS, blue), and transposons (Tn, green). **(b)** Specialist versus generalist mobilization profiles of MGEs, based on the number of carbapenemase genes significantly associated with each element. Each bubble represents an MGE, with size proportional to the number of co-occurrences. Most MGEs behave as specialists, but three IS elements (IS6_292, IS91_50, and IS30_173) emerge as extreme generalists, linking to 10–13 distinct carbapenemase genes. **(c)** Network topology of carbapenemase-MGE associations. Nodes represent carbapenemase genes (grey) and MGEs (colored by category); node size is scaled by PageRank centrality. IS6_292 acts as the primary network hub (degree = 13, PageRank = 0.024), connecting multiple carbapenemase families and forming a critical bridge in the dissemination network. **(d)** Comparative mobilization strength among MGE categories. Violin plots show the distribution of log₁₀(odds ratio) values for In, IS, and Tn. Integrons (median OR = 107.2) and transposons (median OR = 92.7) show higher mobilization strength than IS (median OR = 17.3). Statistical comparisons: In vs. IS (^****^p < 10^−6^); IS vs. Tn (^**^p < 0.01); In vs. Tn (ns).

Among the top 30 associations (Figure 5a), the aac(6′)-Iae + INTEGRALL-aacA28 integron partnership showed an odds ratio of 717.041 (FDR = 3.66 × 10^−32^), while dfrA27 co-occurred with its cognate integron cassette at OR = 702.763 (FDR <10^−300^). Notably, it dominated with high mobilization strengths, claiming 19 of the top 30 positions. IS4_84, carrying the aminoglycoside resistance gene *aph*(3′)*-IIa*, emerged as the most potent IS-mediated association (OR = 94.153, rank 8), demonstrating that although IS actively participates in mobilization, it operates at characteristically lower association magnitudes than integron-based systems. This classification revealed a clear mechanistic hierarchy: Integrons specializes in ultra-resistant and cassette-specific mobilization, while IS facilitates broader, but statistically weaker, associations.

We next interrogated whether MGEs exhibit specialist versus generalist strategies in carbapenemase gene mobilization. Classification of 102 MGEs by their association breadth revealed a striking polarization (Figure 5b). Most elements (63/102, 61.8%) operated as specialists, forming significant associations with only one carbapenemase gene. However, a small cadre of three IS elements, IS6_292, IS91_50, and IS30_173, exhibited extreme generalist behavior, each associating with 10–13 distinct carbapenemase genes. IS6_292 emerged as the apex generalist, maintaining 13 significant associations while still preserving high individual association strength (maximum OR = 83.6 with IMP-6). This generalist behavior appeared exclusive to IS; no In or Tn achieved associations with eight or more carbapenemase genes. The specialist-generalist dichotomy was not evenly distributed across MGE categories: IS accounted for all three generalists and showed the highest proportion of moderate-breadth elements (9/45, 20.0%), while In and Tn were predominantly specialist (27/34 and 14/23, respectively). This fundamental difference in mobilization strategy, between high-fidelity specialists and IS as generalists, has profound implications for predicting dissemination routes and designing surveillance strategies.

Network topology analysis identified critical bridge elements whose removal would fragment the carbapenemase mobilome (Figure 5c). Among 159 network nodes (90 carbapenemase genes and 69 MGEs), 10 IS elements dominated the highest betweenness centrality positions, with IS6_292 exhibiting the most extreme hub properties (betweenness = 3815.87, degree = 13, PageRank = 0.024). This element served as the obligate connection point for multiple carbapenemase families, making it a structural linchpin in the dissemination network. IS30_173 (betweenness = 1656.35) and IS1_316 (betweenness = 1461.15) occupied secondary hub positions, together forming a triumvirate of IS that disproportionately control network connectivity. Transposon-mediated connections, while present, exhibited markedly lower betweenness values (maximum 604.78 for TnPMLUA4), indicating their role as peripheral rather than central connectors. The concentration of hub functionality in three IS elements suggests that targeted disruption or monitoring of these specific sequences could have a disproportionate impact on the global spread of carbapenemase genes.

Direct comparison of association strength across MGE categories revealed profound mechanistic differences in mobilization efficiency (Figure 5d). Integrons elements demonstrated the highest median odds ratio (107.2), with associations concentrated in a narrow, high-potency range (Q₁–Q₃: 27.1–1113.8, *n* = 47). Transposons exhibited comparable median strength (92.7) but with dramatically higher variance (Q₁–Q₃: 14.2–855.5, *n* = 38), indicating a more heterogeneous mobilization landscape. IS elements, despite their numerical dominance (*n* = 125 significant associations), operated at characteristically lower association strength (median OR = 17.3, Q₁–Q₃: 7.1–103.4). Statistical comparison confirmed these differences as highly significant: In versus IS (Wilcoxon test, *p* = 7.50 × 10^−7^, ****), and IS versus Tn (*p* = 4.25 × 10^−3^, **). Critically, In and Tn showed no significant difference in median strength (*p* = 0.329, ns), revealing a bimodal distribution in the mobilome: high-strength, low-promiscuity systems (In and Tn) versus moderate-strength, high-promiscuity systems (IS).

### 3.5 Mobilization element distribution in predominant plasmid replicons

The most frequent Inc groups were IncX3 (*n* = 744), IncL (*n* = 661), IncR (*n* = 658), IncFII(pHN7A8) (*n* = 485), IncFII(K) (*n* = 382), IncC (*n* = 370), IncN (*n* = 363), IncFII (*n* = 310), IncFIB(pQil) (*n* = 264), and repB(R1701) (*n* = 228). In plasmid sequences carrying carbapenemase genes, we investigated the distribution of different mobilization elements across Inc groups. For the analysis, we used unique plasmid sequences and assessed how many sequences within each Inc group contained at least one mobilization element associated with carbapenemase genes. Among the 10 most frequent plasmid Inc groups, we observed distinct patterns in the distribution of mobilization elements. Groups such as IncX3 [IS (*n* = 733, 98.5%), Tn (*n* = 152, 20.4%), In (*n* = 3, 0.4%)], IncR [IS (*n* = 656, 99.7%), Tn (*n* = 132, 20.1%), In (*n* = 7, 1.1%)], repB(R1701) [IS (*n* = 217, 95.2%), Tn (*n* = 80, 35.1%), In (*n* = 2, 0.9%)], IncFII(pHN7A8) [IS (*n* = 485, 100%), Tn (*n* = 5, 1.0%)] and IncFII [IS (*n* = 309, 99.7%), Tn (*n* = 29, 9.4%)] were clearly dominated by IS.

In contrast, IncL showed a balanced distribution between IS (*n* = 615, 93.0%) and Tn (*n* = 623, 94.3%). A similar pattern was observed in IncFII(K) [IS (*n* = 374, 97.9%), Tn (*n* = 285, 74.6%), In (*n* = 4, 1.0%)] and IncFIB(pQil) [IS (*n* = 261, 98.9%), Tn (*n* = 189, 71.6%), In (*n* = 2, 0.8%)]. Finally, IncN and IncC plasmids exhibited greater heterogeneity, including a relatively high proportion of In in IncN [IS (*n* = 338, 93.1%), Tn (*n* = 95, 26.2%), In (*n* = 55, 15.2%)] and a mixed distribution in IncC [IS (*n* = 301, 81.4%), Tn (*n* = 146, 39.5%), In (*n* = 26, 7.0%)].

For each Inc group of plasmids, the three most frequent mobilization elements per category were identified, when available. Not all groups contained three elements per category; in such cases, only the elements present were considered. This analysis identified the predominant elements in each group, enabling us to determine the vectors involved in the dissemination of carbapenem resistance genes (Table 1).

**Table 1:** Distribution of the most frequent mobilization elements (insertion sequences, integrons, and transposons) across major plasmid incompatibility groups.

Category	Mobilization element	Abs. freq.	Rel. freq.
IncX3			
IS	IS6_292	517	69.5%
IS	IS30_173	397	53.4%
IS	IS5_112	376	50.5%
In	INTEGRALL-*bla*_IMP-4_-AF445082	2	0.3%
In	INTEGRALL-*dfrA27*-EU675686	1	0.1%
Tn	Tn125	119	16.0%
Tn	Tn4401a	21	2.8%
Tn	Tn5403	12	1.6%
IncL			
IS	IS4_157	583	88.2%
IS	IS1_316	506	76.6%
IS	IS6_292	23	3.5%
Tn	Tn1999.1	540	81.7%
Tn	Tn4401b	51	7.7%
Tn	Tn2696	20	3.0%
IncR			
IS	IS6_292	513	78.0%
IS	IS481_240	391	59.4%
IS	IS1182_104	285	43.3%
In	INTEGRALL-*bla*_IMP-4_-AF445082	2	0.3%
In	In_Tn6025-GU562437	2	0.3%
In	INTEGRALL-*fosC2*-CP071152	1	0.2%
Tn	Tn4401a	78	11.9%
Tn	Tn1999.1	22	3.3%
Tn	Tn5403	13	2.0%
IncFII(pHN7A8)			
IS	IS6_292	469	96.7%
IS	IS481_240	415	85.6%
IS	IS1182_104	221	45.6%
Tn	Tn125	3	0.6%
Tn	Tn2	2	0.4%
IncFII(K)			
IS	IS1182_104	252	66.0%
IS	IS6_292	93	24.3%
IS	IS5_222	76	19.9%
In	INTEGRALL-*bla*_IMP-4_-AF445082	1	0.3%
In	INTEGRALL-*dfrA27*-EU675686	1	0.3%
In	INTEGRALL-*gcu*165-KY883660	1	0.3%
Tn	Tn4401a	253	66.2%
Tn	Tn5403	12	3.1%
Tn	Tn1999.1	9	2.4%
IncC			
IS	IS6_292	123	33.2%
IS	IS91_50	121	32.7%
IS	IS30_173	82	22.2%
In	INTEGRALL-*bla*_VIM-1_-Y18050	11	3.0%
In	INTEGRALL-*smr2*-AJ784256	7	1.9%
In	INTEGRALL-*bla*_IMP-4_-AF445082	3	0.8%
Tn	Tn125	99	26.8%
Tn	Tn4401a	21	5.7%
Tn	Tn5086	16	4.3%
IncN			
IS	IS6_292	209	57.6%
IS	IS1182_104	131	36.1%
IS	IS481_240	48	13.2%
In	INTEGRALL-*bla*_IMP-4_-AF445082	41	11.3%
In	INTEGRALL-*gcu52*-AB616660	8	2.2%
In	INTEGRALL-*smr2*-AJ784256	2	0.6%
Tn	Tn4401b	36	9.9%
Tn	Tn5403	30	8.3%
Tn	Tn4401a	18	5.0%
IncFII			
IS	IS6_292	297	95.8%
IS	IS91_50	283	91.3%
IS	IS30_173	171	55.2%
Tn	Tn2	20	6.5%
Tn	Tn1999.1	3	1.0%
Tn	Tn4401a	2	0.6%
IncFIB(pQil)			
IS	IS1182_104	176	66.7%
IS	IS6_292	71	29.6%
IS	IS30_173	54	20.5%
In	INTEGRALL-*bla*_IMP-4_-AF445082	1	0.4%
In	In36-AY259085	1	0.4%
Tn	Tn4401a	185	70.1%
Tn	Tn4401b	2	0.8%
Tn	Tn125	1	0.4%
repB(R1701)			
IS	IS6_292	146	64.0%
IS	IS1182_104	121	53.1%
IS	IS481_240	112	49.1%
In	INTEGRALL-*bla*_IMP-4_-AF445082	1	0.4%
In	INTEGRALL-*dfrA27*-EU675686	1	0.4%
Tn	Tn4401a	29	12.7%
Tn	Tn5403	22	9.6%
Tn	Tn7247	10	4.4%

For each group, the table reports the three most prevalent elements per category, when available, along with their absolute frequency (Abs. freq.) and relative frequency (Rel. freq.), calculated as the proportion of unique plasmid sequences in the group carrying the element.

### 3.6 Supervector potential across plasmid incompatibility groups

A co-occurrence pattern analysis was performed to identify plasmids with high mobilization potential, defined as “supervectors.” The most representative groups in terms of plasmid number were IncX3 (744 plasmids), IncL (661 plasmids), and IncR (658 plasmids). The mobilization-resistance score allowed us to classify replicons with a high capacity for gene acquisition and dissemination (defined as the product of the number of unique mobilization elements by the number of unique carbapenemase genes), displayed differences between the groups. IncFIB(pQil) had the highest average score (≈2.89), indicating that, on average, its plasmids have greater diversity of genes and mobilization elements. Conversely, IncR had the highest maximum score (22), indicating that this group contains individual plasmids with a very high potential for disseminating carbapenemase genes, although the group’s average score is lower than that of IncFIB(pQil).

Other groups, such as IncFII(pHN7A8), IncFII(K), and IncX3, also presented high mean indices (≈2.59–2.70), combining a relatively high plasmid frequency with good supervector potential. In contrast, groups such as IncC and IncN presented lower mean indices (≈2.20), indicating lower diversity of genes and mobilization elements per plasmid, despite having a considerable number of plasmids (see Table 2).

**Table 2:** Mobilization-resistance score across plasmid incompatibility groups.

Inc group	Plasmids (*n*)	Average score	Max score
IncX3	744	2,700,268,817	10
IncL	661	2,721,633,888	4
IncR	658	2,761,398,176	22
IncFII(pHN7A8)	485	2,593,814,433	10
IncFII(K)	382	2,688,481,675	8
IncC	370	2,213,513,514	8
IncN	363	2,201,101,928	10
IncFII	310	2,712,903,226	8
IncFIB(pQil)	264	2,893,939,394	12
repB(R1701)	228	2,583,333,333	8

Distribution of plasmid incompatibility groups (Inc—incompatibility) carrying carbapenemase genes, showing the number of plasmids in each group (Num. plasmids), the average mobilization-resistance score (average score), and the maximum mobilization-resistance score value observed in each unique plasmid (max Ind. supervector).

## 4 Discussion

Plasmids are the primary vectors for the spread of carbapenem resistance; however, their interaction with other MGEs further increases the mobility of these determinants and makes resistance containment even more complex (Acman et al., 2022). In this study, we analyzed a total of 72,556 publicly available complete plasmid genomes, including 28,836 that carried antimicrobial resistance genes and 6,017 that carried carbapenemase genes. To our knowledge, this represents the first large-scale, systematic characterization of the interactions between the plasmid mobilome and the carbapenemase resistance gene pool. This large-scale study overcomes the limitations of previous regional surveillance studies or analyses, providing a more limited view of the genetic factors driving global carbapenem resistance (Conlan et al., 2014; Jiang et al., 2020; Reyes et al., 2020).

We observed that the majority of MGEs were directly associated with the mobilization of carbapenem resistance determinants, including 73.4% of IS, 85.3% of Tn, and 71.2% of In (Figure 1a). Indeed, previous studies have demonstrated the involvement of MGEs in the dissemination of resistance genes, indicating that many circulating plasmids contain these structures, acting as enhancers of gene mobilization (Partridge et al., 2018; Das et al., 2022).

Our statistical analysis further corroborated these observations, revealing distinct patterns of interaction among MGEs. Integrons exhibited the strongest associations with carbapenemase genes, followed by transposons and insertion sequences, suggesting a hierarchical contribution of these elements to gene mobilization. This pattern suggests that integrons may serve as primary integration platforms, while transposons and insertion sequences provide the genetic plasticity required for secondary mobilization and horizontal transfer. This fundamental difference in mobilization strategy, with highly skilled specialists versus IS as generalists, has profound implications for predicting dissemination routes and planning surveillance strategies. Among the IS families, IS6, IS91, and IS30 exhibited the broadest interaction spectra with different carbapenemase genes, highlighting their versatility and role as generalist drivers of resistance dissemination, rather than gene-specific mediators. The mobilome architecture thus reveals a paradox: the most structurally critical elements (IS hubs with extreme generalist behavior) do not correspond to the strongest individual associations (integron cassettes with ultra-high odds ratios). This dissociation between network centrality and association magnitude suggests that carbapenemase dissemination operates through two complementary modes: strong specialized transmission via In and Tn, and weak generalist transmission via IS, each contributing differentially to the evolutionary dynamics of resistance dissemination. Understanding this duality is essential to predicting which genetic platforms will drive future carbapenemase dissemination under different selective pressures. Notably, these results also reinforce emerging therapeutic perspectives we have previously discussed in the context of CRISPR-Cas-based strategies designed to selectively disrupt resistance determinants, including carbapenemases, thereby illustrating how mobilome-level understanding and precision-editing technologies can converge to inform innovative approaches against antimicrobial resistance (de Souza et al., 2025b; de Souza et al., 2025c).

### 4.1 Mobilization of the *bla*_KPC_ gene

Tn4401, a member a member of the Tn3 family, remains the most frequent platform responsible for *bla*_KPC_ mobilization (Cuzon et al., 2011; Migliorini et al., 2021) and was frequently associated with both *bla*_KPC-2_ and *bla*_KPC-3_ in our dataset. In contrast to previous reports suggesting isoform-specific Tn4401a and Tn4401b preferences (Migliorini et al., 2021; Fortini et al., 2016), our large-scale analysis revealed a balanced distribution of these variants, with both genes commonly associated with the Tn4401a isoform, reinforcing its continued epidemiological relevance.

Beyond this classical framework, our data highlight an expanded repertoire of mobilization platforms for *bla*_KPC_. In particular, Tn7247 emerged as an alternative transposon associated with *bla*_KPC-2_, supporting recent evidence that dissemination of this gene is no longer restricted to the Tn4401 family. This observation indicates an ongoing diversification of genetic vehicles capable of capturing and propagating *bla*_KPC’s_ (Forero-Hurtado et al., 2023).

Notably, a substantial fraction of *bla*_KPC-2_ occurrences were embedded within compound transposition contexts mediated by IS6 family elements (Figure 3b), independent of Tn4401-associated structures. These IS6-mediated pseudo-compound transposons represent a flexible mobilization strategy, enabling the capture and rearrangement of resistance loci through cointegration-driven mechanisms rather than classical transposition (Harmer and Hall, 2019; Harmer et al., 2020). The prominence of IS6 in these configurations underscores its role as a generalist driver of *bla*_KPC_ dissemination, complementing the high-fidelity but more restricted mobilization mediated by dedicated transposons. Collectively, these findings demonstrate that the contemporary spread of *bla*_KPC-2_ is shaped by the interplay between conserved specialist platforms and highly adaptable IS-mediated architectures.

### 4.2 Mobilization of the *bla*_NDM_ gene

The mobilization landscape of *bla*_NDM_ exhibited a distinct evolutionary pattern when compared to other carbapenemase genes. Consistent with previous genomic studies, Tn125 remained the predominant platform associated with *bla*_NDM-1_, supporting its role as the ancestral transposon responsible for the emergence and early dissemination of this gene (Poirel et al., 2012; Bogaerts et al., 2012; Partridge and Iredell, 2012; Acman et al., 2022). Nevertheless, accumulating evidence indicates that this classical architecture is no longer exclusive.

In our dataset, compound transposition contexts involving blaNDM-1 were frequently mediated by insertion sequences, with IS91 emerging as the main flanking element associated with these events (Figure 3b). This observation is consistent with recent reports describing a progressive replacement of Tn125-derived structures by IS-mediated pseudocomposite transposons, which confer increased architectural flexibility to resistance loci (Acman et al., 2022).

A clear divergence was observed between *bla*_NDM_ variants. While *bla*_NDM-1_ retained strong associations with Tn125-related contexts, the *bla*_NDM-5_ variant was predominantly linked to the Tn2 transposon, a well-established mobilization platform historically associated with *β*-lactamase genes (Bailey et al., 2011). In addition, *bla*_NDM-5_ frequently co-occurred with multiple IS families, including IS6, IS30, and IS91, consistent with previous observations that IS-mediated rearrangements facilitate the adaptation of resistance genes to diverse plasmid backbones (Lee et al., 2016; Varani et al., 2021; Harmer and Hall, 2024).

Together, these findings support an evolutionary transition in *bla*_NDM_ mobilization, from an early reliance on a single specialized transposon toward increasingly IS-driven and modular dissemination strategies. This shift toward greater structural plasticity likely contributes to the rapid global expansion and epidemiological success of contemporary *bla*_NDM_ variants.

### 4.3 Mobilization of the *bla*_OXA-48_ gene

The dissemination of *bla*_OXA-48_ displayed a highly conserved mobilization architecture. Consistent with previous reports, Tn1999.1 emerged as the predominant transposon associated with this gene, reinforcing its role as the principal vehicle driving the global spread of *bla*_OXA-48_ among plasmids and across diverse bacterial hosts (Li et al., 2022; Pitout et al., 2019; Peirano and Pitout, 2025).

In our dataset, *bla*_OXA-48_ showed an exceptionally strong and specific association with IS4 family elements, reflected by a Jaccard index of 0.95, underscoring the near-obligate linkage between this resistance determinant and Tn1999-derived structures. This high degree of structural conservation contrasts sharply with the more heterogeneous mobilization patterns observed for other carbapenemase genes.

Beyond the canonical Tn1999.1 platform, we identified Tn2696 as an alternative mobilization structure associated with *bla*_OXA-48_. Originally described in *Escherichia coli* isolates from Italy, Tn2696 represents a derivative of Tn1999 in which the *bla*_OXA-48_ gene is flanked by IS elements of the IS1 family rather than IS4 (Giani et al., 2012). Although less frequent, the presence of this variant highlights that, despite its overall structural conservation, *bla*_OXA-48_ can be accommodated within distinct but closely related composite transposon architectures.

### 4.4 High-mobility plasmids: the supervectors in the propagation of carbapenemase genes

We defined “supervectors” as plasmids with a high mobilization potential, characterized by the presence of multiple carbapenemase genes associated with various MGEs. These elements act synergistically, enabling recombination, integration, and horizontal transfer of resistance determinants, thereby facilitating their dissemination among different bacterial species (Partridge et al., 2018). Although the IncX3, IncL, and IncR Inc groups were the most frequently detected among plasmids carrying carbapenemase genes associated with mobilization elements, the IncFIB(pQil) and IncR plasmids exhibited the highest mean and maximum mobilization-resistance score, indicating superior potential for the spread and acquisition of new resistance determinants.

The high average score observed for IncFIB(pQil) plasmids indicates that they combine a high diversity of mobilization elements with several carbapenemase genes, functioning simultaneously as stable reservoirs and active platforms for gene transfer. The role of these plasmids as supervectors can be explained by two complementary characteristics: (i) their ability to co-integrate with other replicons, forming multireplicon structures, increases the presence of Tn and In, favoring the acquisition and dissemination of multiple resistance genes (Hanke et al., 2024); (ii) their modular plasticity allows them to rearrange genes, integrate new elements, and interact with other plasmids, increasing their adaptability and persistence in different contexts, including hospital settings under high selective pressure from carbapenem use (Wang et al., 2021). These factors make IncFIB(pQil) plasmids highly efficient in propagating critical resistance genes, such as *bla*_KPC_, contributing to the emergence and maintenance of multidrug-resistant strains in clinical settings.

Interestingly, although less prevalent, IncR plasmids exhibited a genetic profile consistent with supervectors frequently associated with *bla*_NDM_ and *bla*_KPC_ genes. Previous studies have demonstrated a strong association between IncR and *bla*_KPC-2_, suggesting its role as a preferential platform for the dissemination of this variant among different carbapenem-resistant bacteria (Feng et al., 2019). Despite lacking a complete conjugation system and therefore not being inherently self-transmissible, IncR plasmids often coexist with other conjugative or mobilizable plasmids, which enables their transfer through cointegration or plasmid-assisted mobilization (Calia et al., 2022). The identification of plasmids that can act as supervectors in the dissemination of carbapenemases demonstrates that the importance of the replicon is not limited to the prevalence of specific Inc groups, but rather to the modular complexity of plasmids, the coexistence of multiple mobilization elements, and their ability to interact with conjugative systems of different origins.

### 4.5 Limitations

A limitation of this study is the use of a fixed genomic proximity threshold (≤5 kb) to define functional AMR-MGE associations. While this conservative criterion was intentionally adopted to minimize possible associations driven by simple co-residence on plasmid backbones, it may underestimate biologically meaningful mobilization events involving larger genetic architectures. In particular, composite transposons, nested transposition units, or plasmid-level mobilization processes may enable the co-dissemination of carbapenemase genes and their mobilization machinery without satisfying a short-range proximity definition. Consequently, our analytical framework is biased toward detecting tightly linked mobilization events and may undercount broader transposon- or plasmid-mediated dissemination structures.

Despite the large scale and global scope of this analysis, we acknowledge that our reliance on publicly available complete plasmid genomes may introduce representational bias. Curated plasmid databases such as PLSDB are inherently enriched for plasmids derived from clinical and surveillance-focused studies, while plasmids circulating in environmental, agricultural, and non-clinical reservoirs remain comparatively underrepresented. This bias reflects current sequencing priorities rather than a limitation of the analytical framework itself and is common to most large-scale plasmid-centered studies. Consequently, the mobilome architectures described here should be interpreted primarily in the context of well-characterized plasmids, particularly those associated with human and animal health.

Importantly, emerging metagenomic approaches targeting plasmid-associated sequences, often referred to as the plasmidome or metaplasmidome, offer promising avenues to overcome current database limitations. Recent studies applying long-read metagenomics and graph-based reconstruction have revealed extensive and previously inaccessible plasmid diversity across non-clinical ecosystems, including the human gut and environmental reservoirs (Yu et al., 2024; Debroas, 2025). Integrating such approaches with plasmid-centric analyses may allow future studies to evaluate whether the specialist versus generalist mobilome architecture identified here also governs carbapenemase dissemination outside clinical settings, or whether distinct dissemination regimes operate in environmental reservoirs.

## 5 Conclusion

This study reveals that the dissemination of carbapenem resistance is driven not by isolated elements, but by a coordinated mobilome, in which plasmids, transposons, integrons, and insertion sequences act together to build highly efficient genetic platforms for the spread of carbapenemases. By mapping these interactions at a large scale, we identified high-risk plasmid replicons, particularly IncFIB(pQil) and IncR, that function as major genetic hubs capable of concentrating multiple MGEs and diverse carbapenemase genes.

Our findings demonstrate that carbapenemase expansion depends on both the structural specialization of certain MGEs and the broad ecological mobility of IS families, reshaping current understanding of how resistance evolves and circulates globally. These insights provide a practical framework for strengthening genomic surveillance, refining intervention priorities, and supporting the development of strategies aimed at disrupting key vectors of resistance transmission.

By clarifying the genetic architecture that sustains the carbapenem resistome, this work provides a foundation for future approaches, including precision tools such as CRISPR-Cas, to target the most responsible markers for driving the spread of carbapenem resistance.

## Data Availability

The original contributions presented in the study are included in the article/Supplementary material, further inquiries can be directed to the corresponding author.

## Footnotes

1
https://tncentral.ncc.unesp.br/

2
https://github.com/tseemann/abricate
