# Supplementary material for: Large-scale plasmidome and carbapenem mobilome analysis reveals a mechanistic duality: high-power specialists and structural generalists mobile genetic elements
Source: Front Microbiol. 2026 Feb 20;17:1755819. doi: 10.3389/fmicb.2026.1755819 (PMC12963056; doi:10.3389/fmicb.2026.1755819)
Supplement: Supplementary file 1 [file Data_Sheet_1.DOCX]

library(readxl)

library(dplyr)

library(writexl)

#--------------------------------------------------

# 1. Ler os arquivos

#--------------------------------------------------

abr <- read_excel("ABRicate_MGE.xlsx") %>%

transmute(

SEQUENCE = SEQUENCE,

START = START,

END = END,

STRAND = STRAND,

GENE = GENE,

Source = "ABRicate"

)

ise <- read_excel("ISEScan_MGE.xlsx") %>%

transmute(

SEQUENCE = SEQUENCE,

START = START,

END = END,

STRAND = STRAND,

GENE = GENE,

Source = "ISEScan"

)

#--------------------------------------------------

# 2. Unir tabelas

#--------------------------------------------------

combined <- bind_rows(abr, ise)

#--------------------------------------------------

# 3. Identificar elementos detectados por ambas

# ferramentas (mesma sequência e coordenadas)

#--------------------------------------------------

final_table <- combined %>%

group_by(SEQUENCE, START, END, STRAND, GENE) %>%

mutate(

Source = ifelse(n() > 1, "Both", Source)

) %>%

distinct() %>%

ungroup()

#--------------------------------------------------

# 4. Exportar tabela suplementar

#--------------------------------------------------

write_xlsx(

final_table,

"Supplementary_Table_S7_Merged_MGE_Annotations.xlsx"

)
